# Supplementary material for: Insulin Resistance in Mitochondrial Diabetes
Source: Biomolecules. 2023 Jan 7;13(1):126. doi: 10.3390/biom13010126 (PMC9855690; doi:10.3390/biom13010126)
Supplement: Supplementary file 1 [file biomolecules-13-00126-s001.zip › biomolecules-1936660-supplementary.pdf]

Table S1. Confirmed pathogenic mtDNA mutations available from MITOMAP. (Accessed on 3 August 2022).

Index	Locus Type	Locus	Associated Diseases	Allele	Position	aaΔ or RNA
1	tRNA	MT-TF	MELAS / MM & EXIT	m.583G>A	583	tRNA Phe
2	tRNA	MT-RNR1	DEAF	m.1494C>T	1494	12S rRNA
3	tRNA	MT-RNR1	DEAF; autism spectrum intellectual disability; possibly antiatherosclerotic	m.1555A>G	1555	12S rRNA
4	tRNA	MT-TV	AMDF	m.1606G>A	1606	tRNA Val
5	tRNA	MT-TV	MNGIE-like disease / MELAS	m.1630A>G	1630	tRNA Val
6	tRNA	MT-TL1	MELAS / Leigh Syndrome / DMDF / MIDD / SNHL / CPEO / MM / FSGS / ASD / Cardiac+multi-organ dysfunction	m.3243A>G	3243	tRNA Leu (UUR)
7	tRNA	MT-TL1	MM / MELAS / SNHL / CPEO	m.3243A>T	3243	tRNA Leu (UUR)
8	tRNA	MT-TL1	MELAS; possible atherosclerosis risk	m.3256C>T	3256	tRNA Leu (UUR)
9	tRNA	MT-TL1	MELAS / Myopathy	m.3258T>C	3258	tRNA Leu (UUR)
10	tRNA	MT-TL1	MMC / MELAS	m.3260A>G	3260	tRNA Leu (UUR)
11	tRNA	MT-TL1	MELAS / DM	m.3271T>C	3271	tRNA Leu (UUR)
12	tRNA	MT-TL1	PEM / retinal dystrophy in MELAS	m.3273delT	3273	tRNA Leu (UUR)
13	tRNA	MT-TL1	Myopathy	m.3280A>G	3280	tRNA Leu (UUR)
14	tRNA	MT-TL1	MELAS / Myopathy / Deafness+Cognitive Impairment	m.3291T>C	3291	tRNA Leu (UUR)
15	tRNA	MT-TL1	MM	m.3302A>G	3302	tRNA Leu (UUR)
16	tRNA	MT-TL1	MMC	m.3303C>T	3303	tRNA Leu (UUR)
17	Coding	MT-ND1	LHON MELAS overlap	m.3376G>A	3376	E24K
18	Coding	MT-ND1	LHON	m.3460G>A	3460	A52T
19	Coding	MT-ND1	LHON	m.3635G>A	3635	S110N
20	Coding	MT-ND1	Progressive Encephalomyopathy / Leigh Syndrome / Optic Atrophy	m.3890G>A	3890	R195Q
21	Coding	MT-ND1	EXIT+myalgia / severe LA+cardiac / 3-MGA aciduria / nephropathy+deafness+diabetes	m.3902_3908 ACCTTGCinv	3902	DLA-GKV
22	Coding	MT-ND1	LHON / Leigh-like phenotype	m.4171C>A	4171	L289M
23	tRNA	MT-TI	CPEO / MS	m.4298G>A	4298	tRNA Ile
24	tRNA	MT-TI	MICM	m.4300A>G	4300	tRNA Ile
25	tRNA	MT-TI	CPEO	m.4308G>A	4308	tRNA Ile
26	tRNA	MT-TQ	Encephalopathy / MELAS	m.4332G>A	4332	tRNA Gln
27	tRNA	MT-TM	Myopathy / MELAS / Leigh Syndrome	m.4450G>A	4450	tRNA Met
28	tRNA	MT-TW	Mitochondrial myopathy	m.5521G>A	5521	tRNA Trp
29	tRNA	MT-TW	Leigh Syndrome	m.5537_5538insT	5537	tRNA Trp
30	tRNA	MT-TA	Myopathy	m.5650G>A	5650	tRNA Ala
31	tRNA	MT-TN	CPEO+ptosis+proximal myopathy	m.5690A>G	5690	tRNA Asn
32	tRNA	MT-TN	CPEO / MM	m.5703G>A	5703	tRNA Asn
33	tRNA	MT-TN	Multiorgan failure / myopathy	m.5728T>C	5728	tRNA Asn
34	Coding	MT-CO1	SNHL	m.7445A>G	7445	term514term
35	tRNA	MT-TS1 precursor	SNHL	m.7445A>G	7445	tRNA Ser (UCN) precursor
36	tRNA	MT-TS1	PEM / AMDF / Motor neuron disease-like	m.7471_7472insC	7471	tRNA Ser (UCN)

37	tRNA	MT-TS1	MM / EXIT	m.7497G>A	7497	tRNA Ser (UCN)
38	tRNA	MT-TS1	SNHL	m.7510T>C	7510	tRNA Ser (UCN)
39	tRNA	MT-TS1	SNHL/Deafness	m.7511T>C	7511	tRNA Ser (UCN)
40	tRNA	MT-TK	Severe adult-onset multisymptom myopathy / Myoclonic epilepsy	m.8306T>C	8306	tRNA Lys
41	tRNA	MT-TK	MNGIE / Progressive mito cytopathy	m.8313G>A	8313	tRNA Lys
42	tRNA	MT-TK	Myopathy / Exercise Intolerance / Eye disease+SNHL	m.8340G>A	8340	tRNA Lys
43	tRNA	MT-TK	MERRF; Other - LD / Depressive mood disorder / leukoencephalopathy / HiCM	m.8344A>G	8344*	tRNA Lys
44	tRNA	MT-TK	MERRF	m.8356T>C	8356	tRNA Lys
45	tRNA	MT-TK	MICM+DEAF / MERRF / Autism / Leigh Syndrome / Ataxia+Lipomas	m.8363G>A	8363	tRNA Lys
46	Coding	MT-ATP6	Mitochondrial myopathy, lactic acidosis and sideroblastic anemia (MLASA) / IgG nephropathy	m.8969G>A	8969	S148N
47	Coding	MT-ATP6	MIDD, renal insufficiency	m.9155A>G	9155	Q210R
48	Coding	MT-ATP6	Leigh Disease / Spastic Paraplegia / Spinocerebellar Ataxia	m.9176T>G	9176	L217R
49	Coding	MT-ATP6	Leigh Disease / Ataxia syndromes / NARP-like disease / Episodic weakness and Charcot-Marie-Tooth	m.9185T>C	9185	L220P
50	Coding	MT-ATP6	Encephalopathy / Seizures / Lacticacidemia	m.9205_9206delTA	9205	Ter-M
51	tRNA	MT-TG	PEM	m.10010T>C	10010	tRNA Gly
52	Coding	MT-ND3	Leigh Disease / Leigh-like Disease / ESOC	m.10191T>C	10191	S45P
53	Coding	MT-ND3	Leigh Disease / Dystonia / Stroke / LDYT	m.10197G>A	10197	A47T
54	Coding	MT-ND4	Leigh Disease	m.11777C>A	11777	R340S
55	Coding	MT-ND4	LHON / Progressive Dystonia	m.11778G>A	11778	R340H
56	tRNA	MT-TH	MERRF-MELAS / Encephalopathy	m.12147G>A	12147	tRNA His
57	tRNA	MT-TH	Maternally inherited non-syndromic deafness	m.12201T>C	12201	tRNA His
58	tRNA	MT-TS2	DMDF / RP+SNHL	m.12258C>A	12258	tRNA Ser (AGY)
59	tRNA	MT-TL2	CPEO	m.12276G>A	12276	tRNA Leu (CUN)
60	tRNA	MT-TL2	CPEO / EXIT+Ophthalmoplegia	m.12294G>A	12294	tRNA Leu (CUN)
61	tRNA	MT-TL2	CPEO / KSS / possible carotid atherosclerosis risk, trend toward myocardial infarction risk	m.12315G>A	12315	tRNA Leu (CUN)
62	tRNA	MT-TL2	CPEO	m.12316G>A	12316	tRNA Leu (CUN)
63	Coding	MT-ND5	Leigh Disease	m.12706T>C	12706	F124L
64	Coding	MT-ND5	LHON	m.13051G>A	13051	G239S
65	Coding	MT-ND5	Ataxia+PEO / MELAS, LD, LHON, myoclonus, fatigue	m.13094T>C	13094	V253A
66	Coding	MT-ND5	LHON	m.13379A>G	13379	H348R
67	Coding	MT-ND5	Leigh Disease / MELAS / Ca2+ downregulation	m.13514A>G	13514	D393G
68	Coding	MT-ND6	MELAS / Leigh Disease	m.14453G>A	14453	A74V
69	Coding	MT-ND6	LHON	m.14482C>A	14482	M64I
70	Coding	MT-ND6	LHON	m.14482C>G	14482	M64I
71	Coding	MT-ND6	LHON	m.14484T>C	14484	M64V
72	Coding	MT-ND6	Dystonia / Leigh Disease / ataxia / ptosis / epilepsy	m.14487T>C	14487	M63V
73	Coding	MT-ND6	LHON	m.14495A>G	14495	L60S
74	Coding	MT-ND6	LHON	m.14568C>T	14568	G36S
75	tRNA	MT-TE	Reversible COX deficiency myopathy	m.14674T>C	14674	tRNA Glu

76	tRNA	MT-TE	MM+DMDF / Encephalomyopathy / Dementia+diabetes+ophthalmoplegia	m.14709T>C	14709	tRNA Glu
77	tRNA	MT-TE	Encephalomyopathy + Retinopathy	m.14710G>A	14710	tRNA Glu
78	Coding	MT-CYB	EXIT / Septo-Optic Dysplasia	m.14849T>C	14849	S35P
79	Coding	MT-CYB	Multisystem Disorder, EXIT	m.15579A>G	15579	Y278C
80	tRNA	MT-TP	MM / PEO	m.15990C>T	15990	tRNA Pro
